# Editorial—Special Issue on “Optical and RF Atmospheric Propagation”

**DOI:** 10.3390/s23073644

**Published:** 2023-03-31

**Authors:** Natan Kopeika, A Arockia Bazil Raj

**Affiliations:** 1 Department of Electro-Optics and Photonics Engineering, School of Electrical and Computer Engineering, Ben-Gurion University of the Negev, Beer Sheva 84105, Israel; 2 Electronics Engineering, Defence Institute of Advanced Technology, Pune 411025, India

## 1. Introduction

This Special Issue presents the latest research and developments in the field of optical and RF propagation sensing, propagation/effects/channel molding, advancements in applications, signal far-field measurements, theoretical/measurement methods for beam handling/processing, military applications, and next-generation network formations, amongst others. As sensing and measurements are the primary requirements for all the above fields, these topics thus fall within the scope of *Sensors*. An application common to both optical and RF atmospheric propagation is imaging. An advantage in the spectral region is the ability to image objects concealed in or by non-conducting media such as clothing, luggage, and various dielectric containers. The disadvantages include the relatively poor image quality caused by diffraction, relatively large pixel size, and the relatively high cost of millimeter wave (MMW) components, especially detectors, compared to optical ones. On the other hand, atmospheric transmission is usually much better than in optical spectral regions.

## 2. Brief on Research Articles Published

In [1], we present an update on the use of very inexpensive neon plasma indicator lamp glow discharge devices (GDD), which cost about half a dollar (USD) each, as detector pixels for MMW imaging. In this article, a new design for a row detector using GDDs as pixel elements and the influence of MMW incidence on GDD’s discharge current were acquired using an elementary data acquisition (DAQ) platform. The DAQ system computes the averaged Fast Fourier Transform (FFT) spectrum of the time signal and returns the FFT results as a magnitude based on the level of detection. An FFT-based signal acquisition proved to be a better alternative to the lock-in detection commonly used in MMW detection systems. This improved detection circuit provides enhanced noise filtering, thereby resulting in better MMW images within a short time. The overhead expense of the entire system is very low, as it can avoid lock-in amplifier stages that were previously used for signal enhancement. A scanning mechanism using a motorized translation stage (step motor) is involved to place and align the row detector in the image plane. The scanning can be carried out vertically to perform imaging by configuring the step motor after selecting the desired step size and position. A simplified version of the MMW detection circuit with a dedicated over-voltage protection facility is presented here, improving the stability and reliability of the detection system during its operation. The MMW detection circuit demonstrated in this work was found to be a possible milestone to develop larger focal plane arrays with very inexpensive sensor elements. Improved image quality deriving from oversampling is demonstrated for very small focal plane arrays.

It is well known that when landing or taking off with propellor-driven aircraft, such as helicopters, in dry sand regions, such as deserts or coastal beaches, the helicopter propellor motion can give rise to enormous quantities of dry sand that are uplifted by the propellor motion to such an extent that visibility is essentially zero. This situation is extremely dangerous, and accidents can be frequent. This phenomenon is known as “brownout”. Unlike visible and infrared systems, the radar devices in the microwave or millimeter wave region offer the capability of sufficient transmission through atmospheric obscurants, such as fog, smoke, sand/dust storms, and brownout. In [2], we present a theoretical evaluation of MMW (85–100 GHz) attenuation/scattering and power transfer in brownout conditions. The model includes attenuation/scattering prediction and radiant flux, or power collected by the receiver. This paper considers the case of sand grain clouds created by helicopter rotor airflow during landing in arid areas. The evaluated scenarios are brownout environments over ranges up to 50 m. The predicted values from the mathematical model are compared with findings in the field and the literature. A simple model for mm wave power transfer estimation shows satisfactory agreement with the measured values. 

Greenhouse gas (GHG) emissions from rice fields have huge effects on climate change. Low-cost systems and management practices that can quantify and reduce GHGs emission rates are needed to improve climate conditions. The typical GHGs estimation processes are expensive and depend mainly on high-cost laboratory equipment. In [3], a low-cost sensor-based GHG sampling and estimation system for rice fields is presented. For this, a fully automatic gas chamber with a sensor-integrated gas accumulator and quantifier unit was designed and implemented to study its performance in the estimation efficiency of greenhouse gases (CH_4_, N_2_O, and CO_2_) from rice fields for two crop seasons. For each crop season, three paddy plots were prepared at the experimental site and then subjected to different irrigation methods (continuous flooding (CF), intermittent flooding (IF), and controlled intermittent flooding (CIF)) and fertilizer treatments to study the production and emission rates of GHGs at regular intervals throughout the crop growing season. A weather station was installed on the site to record the seasonal temperature and rainfall events. The seasonal total CH_4_ emission was affected by the effects of irrigation treatments and the mean CH_4_ emission in the CIF field was smaller than in other treatments. CH_4_ and N_2_O emission peaks were high during the vegetative and reproductive phases of rice growth, respectively. The results indicated that CIF treatment is most suitable in terms of rice productivity and higher water use efficiency. The application of nitrogen fertilizers produced some peaks in N_2_O emissions. On the whole, the proposed low-cost GHGs estimation system performed well during both crop seasons, and it was found that the adaption of CIF treatment in rice fields could significantly reduce GHG emissions and increase rice productivity. The research results also suggested some mitigation strategies that could reduce the production of GHGs from rice fields.

In [4], the C-band (5.3 GHz) radiofrequency (RF) sensing is considered for multiple low radar cross-section (RCS) aerial targets. The popularity of low RCS targets is increasing day by day, and the detection and identification of these targets have become critical issues. micro-Doppler signatures are key components for detecting and identifying these low RCS targets. For this, an innovative approach is proposed along with the smooth pseudo Wigner–Ville distribution (SP-WVD) and adaptive filter bank to improve the attenuation of cross-term interferences to generate more accurate images for the micro-Doppler signatures/patterns of simultaneous multiple targets. A C-band sensor is designed and used to acquire the micro-Doppler signatures of aerial rotational, flapping, and motional low RCS targets. Digital pipelined-parallel architecture is designed inside the Xilinx field-programable gate array (FPGA) for fast sensor data collection, data preprocessing, and interface to the computer (imaging algorithm). The experimental results of the proposed approach are validated with the results of the classical short-term Fourier transform (STFT), continuous wavelet transform (CWT), and smooth pseudo Wigner–Ville distribution (SP-WVD). Realistic open-field outdoor experiments are conducted, covering different simultaneous postures of (i) two-/three-blade propeller/roto systems, (ii) flapping bionic bird, and (iii) kinetic warhead targets. The associated experimental results and findings are reported and analyzed here, and limitations and possible future research studies are also discussed in the conclusions.

In [5], continuing with aerial targets, we now proceed from RF to infrared (IR) imaging target acquisition. Atmospheric path radiance in infrared is extremely important in calculating system performance in certain infrared detection systems. For infrared search and track (IRST) system performance calculations, the path radiance competes with the target for precious detector well electrons. In addition, the radiance differential between the target and the path radiance defines the signal level that must be detected. Long-range, high-performance, offensive IRST system design depends on accurate path radiance predictions. In addition, in new applications such as drone detection, where a dim unresolved target is embedded into a path radiance background, sensor design and performance are highly dependent on atmospheric path radiance. Being able to predict the performance of these systems under particular weather conditions and locations has long been an important topic. The authors used MODTRAN, which has been a critical tool in the analysis of systems and the prediction of electro-optical system performance, over many years for an average system performance using the typical “pull-down” conditions in the software. This article considers the level of refinement required for a custom MODTRAN atmosphere profile to satisfactorily model an infrared camera’s performance for a specific geographic location, date, and time. The average difference between a measured sky brightness temperature and a MODTRAN predicted value is less than 0.5 °C with sufficient atmosphere profile updates. The agreement between experimental results and MODTRAN predictions indicates the effectiveness of including updated atmospheric composition, radiosonde, and air quality data from readily available Internet sources to generate custom atmosphere profiles.

In [6], the basic scheme of various first-order optical systems is often arbitrary multiple masks placed at arbitrary positions. Generally, masks in optical systems have a non-shift invariant (SI) effect; thus, the individual effect of each mask on the output cannot be entirely separated. This paper develops a technique where complete separation might be achieved in the common case of random phase screens (RPSs) as masks. RPSs are commonly used to model light propagation through the atmosphere or through biological tissues. The authors demonstrate the utility of the technique on an optical system with multiple RPSs that model random scattering media.

## 3. Conclusions

All the research papers published in this Special Issue address the current topics pertaining to Optical and RF Atmospheric Propagation.

## Data Availability

Not applicable.
